# The administrative costs of community-based health insurance: a case study of the community health fund in Tanzania

**DOI:** 10.1093/heapol/czt093

**Published:** 2013-12-12

**Authors:** Josephine Borghi, Suzan Makawia, August Kuwawenaruwa

**Affiliations:** ^1^London School of Hygiene and Tropical Medicine, 15–17 Tavistock Place, London WC1H 9SH, UK and ^2^Ifakara Health Institute, Kiko Avenue, Plot 463, Kiko Avenue Mikocheni, P.O. Box 78, 373 Dar es Salaam, Tanzania

**Keywords:** Community-based health insurance, administration cost, Tanzania

## Abstract

Community-based health insurance expansion has been proposed as a financing solution for the sizable informal sector in low-income settings. However, there is limited evidence of the administrative costs of such schemes. We assessed annual facility and district-level costs of running the Community Health Fund (CHF), a voluntary health insurance scheme for the informal sector in a rural and an urban district from the same region in Tanzania. Information on resource use, CHF membership and revenue was obtained from district managers and health workers from two facilities in each district. The administrative cost per CHF member household and the cost to revenue ratio were estimated. Revenue collection was the most costly activity at facility level (78% of total costs), followed by stewardship and management (13%) and pooling of funds (10%). Stewardship and management was the main activity at district level. The administration cost per CHF member household ranged from USD 3.33 to USD 12.12 per year. The cost to revenue ratio ranged from 50% to 364%. The cost of administering the CHF was high relative to revenue generated. Similar studies from other settings should be encouraged.

**KEY MESSAGES**

- Advertising and revenue collection were the most resource-intensive activities related to community-based health insurance administration in Tanzania. These activities are done by health workers at the facility and represent a substantial time burden.
- Stewardship and management activities were the most costly activities at district level. These activities were made more time consuming because of a lack of computerized systems for processing membership information for reporting. Pooling and purchasing costs were minimal, due to a lack of risk equalisation or cross subsidisation and limited purchasing.
- The cost of administering the CHF was high relative to revenue generated. The cost to revenue ratio far exceeded the recommended 30%.
- Facilities with lower case loads were able to achieve a lower cost to revenue ratio than facilities with higher case loads meaning that, as currently structured, the CHF lends itself better (in terms of management cost) to small dispensaries than large health centres.

## Introduction

Providing access to affordable health care for those among the informal sector remains a considerable challenge for many low-income countries striving to make progress towards universal health coverage (UHC). As a result there has been considerable debate regarding how countries should reform health financing arrangements to achieve UHC (World Health Organization 2010; Kutzin 2012, 2013; Garrett *et al.* 2012; Evans *et al.* 2012). Community-based health insurance (CBHI) has been promoted as a potential step in the transition towards UHC in low-income countries with a system of user fees at government facilities (World Health Organization 2010; Carrin *et al.* 2008). Indeed, such schemes can enhance access and reduce out-of-pocket payments for those that contribute. However, it is recognised that voluntary contributions cannot achieve UHC, and a substantial reliance on compulsory contributions coupled with subsidies for the poor is a necessary condition for universality (Kutzin 2012).

CBHI can be differentiated from social health insurance in relation to the voluntary nature of contributions (Ekman 2004), its focus on those groups outside the formal sector, and ownership/management by local organizations or groups (providers, local government, NGO, community groups) (Bennett *et al.* 1998; Jakab and Krishnan 2001). CBHI can be differentiated from private health insurance by the not-for-profit nature of its operations (Ekman 2004). The size of the risk pool is also generally limited (International Labour Organization 2002). However, there is wide variation in the design and implementation of such schemes across countries (Carrin *et al.* 2005; Bennett *et al.* 1998).

A number of reviews of the effects of CBHI on health services and populations have been carried out. There is evidence of an overall positive effect of CBHI on service utilisation (Spaan *et al.* 2012; Acharya *et al.* 2012) and financial protection among members (Spaan *et al.* 2012; Acharya *et al.* 2012; Ekman 2004), although very few evaluation studies have adopted experimental or quasi-experimental designs (Ekman 2004; Spaan *et al.* 2012). There is much less evidence on the effects of CBHI on quality of care (Spaan *et al.* 2012) or the efficiency of service delivery (Ekman 2004). A number of studies have explored the effects of CBHI on resource mobilisation (Spaan *et al.* 2012) and cost recovery, with positive effects reported (Ekman 2004; Spaan *et al.* 2012). However, there has been relatively little research into the administrative costs of CBHI schemes in low-income settings and the relative cost of different administrative activities. Yet, it is acknowledged that voluntary insurance schemes typically result in high administrative costs, relative to compulsory schemes (Mathauer and Nicolle 2011). This is due to the bureaucracy required to set premiums and manage funds and costs associated with advertising, card distribution and revenue collection (Mossialos and Thompson 2004). Existing evidence on administrative costs of CBHI from low-income settings is very limited. Such costs have been found to vary between 5% of total scheme revenue in Congo (Bennett *et al.* 1998) to 65% of total scheme expenditure in Rwanda (Mathauer and Nicolle 2011). However, these figures are drawn from national health accounts data or surveys of schemes which provide aggregate data, omitting start-up costs and the value of unpaid staff time which can be substantial. It is also unclear which administrative activities were included in these cost estimates and, in the context of schemes supported by donors, whose costs were included.

As countries make commitments to move towards universal coverage and have to make choices about the appropriate financing mix to meet these objectives, understanding the economic cost of CBHI and the relative cost of different administrative activities is especially pertinent, to ascertain how such schemes can be more efficiently designed, and whether such schemes are financially viable.

Against this background, we set out to measure the administrative costs of the community health fund (CHF), a voluntary community insurance scheme targeting the rural informal sector in Tanzania at the facility and district levels.

### Health insurance in Tanzania

The Tanzanian government is committed to achieving universal coverage. The current target is to increase insurance coverage from 12% to 30% among the population by 2015 (Borghi *et al.* 2013), through expansion of the CHF (Box 1).

**Box 1 Overview of the design and management systems for the CHF**

Design

The Community Health Fund (CHF) is a district-level voluntary insurance scheme targeting the informal sector. Households (a couple and up to eight children) can enrol at primary government health facilities for between USD 3.16–USD 15.82 per year. Benefits include free outpatient care at the facility of registration and referral care in a few districts that have contracted with higher-level facilities. The central government will fully match the contributions made by CHF members through a matching grant. The design of the CHF is specified in a by-law that is approved by district managers, community groups and the Prime Minister’s Office.

Fund management

Primary-level providers are not reimbursed for use of services by CHF members, but facilities can use CHF revenue to purchase drugs, medical supplies, equipment, furniture and undertake maintenance work and pay certain allowances. In some districts there is a central CHF account where the funds are deposited. In others, facilities have their own bank accounts and deposit funds directly.

The Council Health Service Board (CHSB) which comprises medical professionals and community representatives oversees the use of funds from user fees and the CHF. A CHF co-ordinator, who is typically employed as a health secretary, oversees the operation of the CHF, and reports to the district on membership, fund generation and use.

A Ward Health Committee (operating at the ward level, which is the lowest government administrative structure at the community level usually representing between 1000 to 21 000 people) and the Health Facility Governing Committee (HFGC) along with health workers in the facility are responsible for getting people to join, and informing the community about funds collected and funds used. The HFGC members, who are comprised of facility health workers and elected community members, are trained by the CHF co-ordinator.

## Methods

### Study sites

A case-study approach was adopted. Two case-study districts (an urban and a rural district) were selected from the same region. They differed in terms of levels of CHF coverage, duration of CHF implementation and the provision of referral care within the benefit package (Table 1). In the rural district, CHF funds were pooled at the district level in a ‘CHF account’. In the urban district, facilities had their own bank accounts since 2007/2008 where CHF funds were deposited.

**Table 1:** Selected characteristics of sampled case-study districts

Characteristics	Rural district	Urban district
Population size	486 900	175 717
Number of health facilities	57	16
Number of government facilities	50	10
Population per health facility	8542	10 982
Year of introducing CHF	1999	2008
CHF premium	3.16 USD per household per year	3.16 USD per household per year
Total CHF members	9127	683
CHF benefit package	Outpatient care at a selected public primary facility (dispensary or health centre) plus referral care up to 9.49 USD in the regional hospital, or the district designated hospital (faith-based)	Outpatient care at a selected public primary facility (dispensary or health centre)
User fee level	0.63 USD until 2009	0.63 USD for dispensaries and health centres
	1.90 USD since 2009	
Financial flows	CHF and user fee revenue pooled in district CHF account	CHF and user fee revenue deposited in facility bank account

### Cost calculation

We estimated the annual recurrent facility and district-level costs of CHF administration from the perspective of the Ministry of Health and Social Welfare. An economic costing was conducted whereby the value of all inputs required to administer the CHF was included, even if they did not represent an additional financial cost. So, for example, the value of time spent by health workers managing the scheme was valued at their respective salaries even though they are not paid extra to work on the CHF. Capital costs were not included. Average annual administration costs per CHF member household were also estimated along with the administration cost to CHF revenue ratio. The estimation of higher level administrative costs (at regional, zonal and national levels) was outside the scope of the current study. All costs are presented in USD; Tanzanian shillings were converted to dollars using the average annual exchange rate for 2011: Tanzanian shillings 1580 to the dollar (Bank of Tanzania 2012).

### Data sources

An ingredients approach, whereby quantities of each of the inputs are first identified and then prices are attached, was employed to estimate the majority of recurrent costs (salaries, allowances, supplies and transport) (Drummond *et al.* 2005). To identify the main administration activities, associated person time and other resource use, we interviewed the CHF co-ordinator, the health facility in-charge and two health-facility governing committee (HFGC) members from two health facilities in each district (a public health centre and a dispensary). Minutes of quarterly meetings of council health service board (CHSB) and HFGC members were reviewed to determine the number of meetings where the CHF was on the agenda and the duration of these meetings.

Assumptions made during the analysis are outlined in Annex 1 in supplementary data. Unit cost data were derived from national salary scales (United Republic of Tanzania 2007) for personnel and from the CHF co-ordinator (HFGC training costs, and prices of the CHF card), and market prices were used to value other supplies (Annex 2, Table 1A in supplementary data). Information on CHF membership at selected facilities and across the whole district was also obtained along with reports of CHF and matching grant revenue.

### Classification of costs

Costs were classified by resource inputs (salaries, allowances, supplies and transport), and by activity including start-up costs, that is, start-up activities required to introduce the CHF in a district (training HFGC members, purchasing: entering into contract with referral facilities); and ongoing activities using the framework proposed in Mathauer and Nicolle (2011) (revenue collection: advertising and marketing the CHF, registration and enrolment of members; and stewardship: reporting and attending meetings). Only the costs of activities that were clearly related to CHF administration and not to routine service delivery were included. Certain activities were not specific to the CHF. For example, reporting on cost sharing revenue involves reporting on user fee and CHF revenue. This would be done in the absence of the CHF, assuming user fees remain. Costs were estimated with and without these costs as ‘total costs’ (inclusive of joint costs) and ‘pure CHF costs’ (excluding joint costs), respectively.

The average cost per facility and the average district-level management costs are presented. The average annual total district cost was also estimated for the urban and rural district by multiplying the average facility cost by the number of facilities in each of the districts and adding the district level and start-up costs.

### Sensitivity analysis

A two-way sensitivity analysis was conducted to see how the cost per CHF member household varies as a function of facility case load intensity and the number of CHF members registered per facility, holding premiums constant. We also considered how many CHF members would need to register per facility in order that costs do not exceed 30% of revenue, at current premiums. We also explored by how much the premium would need to increase in order for the cost to revenue ratio to fall to 30%, under baseline conditions, and conditions under planned reforms. We estimated the effect of planned reforms, notably the appointment of full time CHF co-ordinators, and the provision of individual instead of household membership cards on annual costs and the cost per CHF member. Given potential uncertainty around health worker reports of time spent advertising CHF within facilities, we also examined the cost to revenue ratio in the absence of such costs.

Ethical clearance for this study was obtained from the Institutional Review Board at the Ifakara Health Institute, and from the World Health Organization.

## Results

### Overview of CHF administration activities

The most significant CHF administration task was informing people about the CHF and advertising and marketing the scheme to try and get people to join (Table 2; Table 2A in supplementary data). To this end health workers reportedly spent a couple of minutes with every uninsured outpatient (Table 2A in supplementary data). Advertising the scheme was also undertaken during routine health promotion talks at facilities. The HFGC members reported spending minimal time on such activities within the communities. The registration and enrolment of CHF members and pooling was also done by health workers. The latter consisted of depositing funds in the bank. Health workers also fulfilled a stewardship and management role, producing monthly and quarterly reports on membership and fund levels. Overall, the time spent by health workers administering the CHF was equivalent to 33% of a single full-time person in urban health centres (23% in rural health centres) and 16% in urban dispensaries (6% in rural dispensaries).

**Table 2:** Overview and description of key CHF administration activities carried out by stakeholders

	Description of tasks	Key stakeholders involved	Number of times reported
**Routine activities**			
**Revenue collection**			
Advertising, marketing: *Individual*	A brief discussion with outpatients about the CHF, with a view to encouraging patients to sign up	All health workers	All facilities
Advertising, marketing: *Group*	Discussion of CHF integrated into weekly health promotion/education discussions with patients at the facility	Health worker	Three out of four facilities
Advertising, marketing: *Community*	Varied including: participation in village meetings; participation in ward-level meetings; ad hoc discussions with community members	Health facility governing committee	Two out of four committees reported attending village meetings
			Two out of four committees reported attending ward meetings
			Three out of four committees reported having ad hoc community discussions
			One committee did not report any activity
Registration and enrolment of members	Recording member details in the cash register, receiving payment, providing a receipt	Health worker	All facilities
**Pooling**			
Pooling	Transfer of funds to district accountant or deposit of funds in facility bank account	Health facility in-charge	All facilities
		District accountant	
**Stewardship and management**			
Meetings: *Facility level*	HFGC meetings to discuss cost sharing revenue and decide on expenditures	HFGC, including facility in-charge	All facilities
	Ward development committee meetings	Ward development committee members and facility in-charge	Three out of four facilities
	Village meetings	Village members and facility in-charge	One out of four facilities
	Facility management team meetings	Facility management team members	One out of four facilities
Meetings: *District level*	Meeting to review CHF membership, cost sharing revenue and approve facility use of revenue	CHSB and CHF co-ordinator	All districts
Reporting: *Facility level*	A report of CHF membership and CHF and user fee revenue is compiled and submitted to the CHF co-ordinator	Facility in-charge, CHF co-ordinator	All facilities
Reporting: *District level*	A report of CHF membership by facility and CHF revenue, user fee revenue is compiled and submitted to the CHSB	CHF co-ordinator	All districts
Supervision	Supervision of CHF during routine Council Health Management Team (CHMT) supervision visits	CHF co-ordinator, CHMT, health workers	Supervision of CHF during CHMT supervision (all districts)
	CHF specific supervision visits to facilities to check CHF records, advertising, marketing and revenue reporting	CHF co-ordinator, health workers	Independent supervision visits (one district)
**One-off start up activities**			
Training	Training of HFGC members on their roles and on CHF	HFGC members and CHF co-ordinator	All districts
Purchasing	Time spent negotiating with district management and in-charges of referral facility in order to establish contracts for service delivery to members	CHF co-ordinator, District Executive Director, CHSB and facility management	One district

At the district level, the main activity was stewardship and management including: facility supervision and compilation of quarterly reports on membership levels and revenue (Table 2; Table 2A in supplementary data). The district CHF co-ordinators spent 3% (urban district) and 18% (rural district) of their time on CHF administration.

### Costs of administering the CHF

The average annual costs of administering the CHF at the facility level were USD 1154, or USD 897 if only the ‘pure’ CHF activities are included (Table 3). Revenue collection constituted the most costly activity at facility level (78%) and especially advertising the CHF (67%), followed by stewardship and management (13%) and pooling of funds (10%). Personnel costs were 85% of the total, followed by the cost of supplies (7%).

**Table 3:** Average annual facility-level costs in USD by input and activity (percentage share of total cost)

	Revenue Collection			Pooling	Stewardship			Total	Pure CHF
	Marketing	Registration	Total	Pooling	Meetings	Reporting	Total		
Salaries	776	42	817	83	22	59	81	981 (85%)	817 (91%)
Allowances	–	–	–	–	65	–	65	65 (6%)	
Transport	–	–	–	27	–	–	–	27 (2%)	
Supplies	–	80	80	1	–	–	–	81 (7%)	80 (9%)
Total	776 (67%)	121 (11%)	897 (78%)	110 (10%)	87 (8%)	59 (5%)	146 (13%)	1154 (100%)	897 (100%)

The average annual costs of stewardship and management of the CHF by district managers were more than 10 times higher in the rural than the urban district (USD 5177 compared to USD 489) (Table 4). The main management costs in the urban district were meetings and report writing. In rural areas, training of facility governing committee members and supervision were substantial costs (Table 4).

**Table 4:** Total annual district-level stewardship and management costs in USD (percentage share of total)

	Urban district			Rural district		
	Salaries	Allowances	Total	Salaries	Allowances	Total
Meetings	30	149	179 (37%)	258	248	507 (10%)
Reporting	172	–	172 (35%)	289	–	289 (6%)
Supervision	28	–	28 (6%)	340	133	473 (9%)
Total recurrent costs	230	149	379 (77%)	887	381	1268 (25%)
Training			110 (23%)			3,762 (73%)
Contracting			-			147 (3%)
**Total start-up costs**			**110 (23%)**			**3908 (75%)**
**Total**	**230**	**149**	**489**	**887**	**381**	**5177**

Total administrative costs across the district (including facility and district management costs) were almost four times higher in the rural than the urban district (USD 28 861 compared with USD 7860). Facility costs dominated in both districts (representing over 90% of total costs in the urban district, and 75–82% in the rural district) (Table 5).

**Table 5:** Total annual district-wide costs in USD by district (percentage share of total)

	Urban district		Rural district		Average	
	Total cost	Total pure	Total cost	Total pure	Total cost	Total pure
Dispensaries	5428	4032	18 157	11 996	11 793	8014
Health centres	1943	1686	5527	3901	3735	2794
Total facility costs	7371 (94%)	5718 (92%)	23 684 (82%)	15 897 (75%)	15 528 (85%)	10 808 (79%)
Total district costs	489 (6%)	489 (8%)	5177 (18%)	5177 (25%)	2833 (15%)	2833 (21%)
Total	7860	6207	28 861	21 074	18 361	13 641

However, average administrative costs per CHF member were over three times higher in the urban than the rural district USD 11.51 per CHF member (USD 9.09 when considering ‘pure’ costs only) compared to USD 3.16 per member (USD 2.31 when considering ‘pure’ costs only) respectively (Table 6). The cost-to-revenue ratio was between three and six times higher in the urban than the rural district (364% or 287% when considering ‘pure’ costs only in the urban district and 100% or 73% when considering ‘pure’ costs only, respectively in the rural district). These estimates exclude matching funds in the estimation of revenue. When matching funds are included in the revenue estimate these figures were 61% in the rural district or 45% when considering ‘pure’ costs only (Table 6).

**Table 6:** Average annual administrative cost per CHF member and cost-to-revenue ratio

	Urban district		Rural district		Average	
	Total cost	Pure CHF	Total cost	Pure CHF	Total cost	Pure CHF
	7860	6207	28 861	21 074	18 361	13 641
Total CHF member households	683		9127		4905	
Total cost per CHF member household (USD)	11.51	9.09	3.16	2.31	3.74	2.78
Total revenue (inclusive matching fund) (USD)	2161 (2161)		28 882 (47 326)		15 522 (24 743)	
Total cost as percentage of revenue (inclusive matching fund)	364% (364%)	287% (287%)	100% (61%)	73% (45%)	118% (74%)	88% (55%)

### Sensitivity analysis

**Figure 1:**
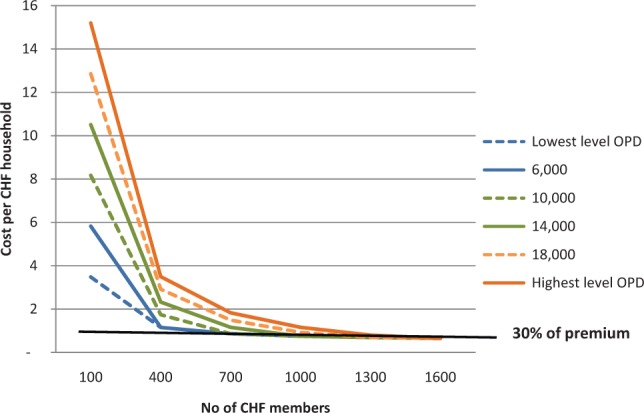
Cost per CHF household in USD as a function of outpatient department visits (OPD) and CHF membership levels

Facilities with lower case load (2000 outpatients per year) would need to attract around 400 CHF member households, in order to reduce costs to 30% of revenue (Figure 1). However, facilities with higher case loads (such as health centres) will struggle to reduce costs below this threshold. Once CHF membership exceeds a certain level, the need to advertise the CHF would theoretically reduce substantially, as all or most outpatients would be covered by insurance. Overall, it is easier for facilities with lower caseload to more rapidly achieve full coverage among their outpatients.

In the rural district, if household premiums were to increase to USD 10.76 per year (USD 7.91 for ‘pure’ costs), the cost to revenue ratio would reduce to 30%, with the current design of CHF (Table 7). However, in the urban district, where membership levels are low, premiums would need to increase to USD 38.61 per year (USD 30.38 for ‘pure’ costs). If district managers become full time CHF co-ordinators and individual cards are distributed, the premium would need to increase by a further USD 6 in the rural district, but by more than double again in the urban district.

**Table 7:** Threshold analysis: premium level required so that the cost to revenue ratio reduces to 30% in USD

	Urban		Rural		Average	
	Total cost	Pure CHF	Total cost	Pure CHF	Total cost	Pure CHF
Baseline	38.61	30.38	10.76	7.91	24.68	19.30
Future design	79.11	71.20	16.77	13.92	47.47	42.41

Even assuming health workers spent no time advertising the CHF, the cost to revenue ratio would still be 215% (79% for ‘pure’ costs) in urban areas and 58% (31% for ‘pure costs’ in rural areas (36% or 19% for ‘pure’ costs if matching grant revenue is included).

If the district manager were to become a full time CHF co-ordinator this would lead to a 19-fold increase in district management costs in the urban district and a 6-fold increase in the rural district (data not shown). If CHF membership cards were given to each individual within the household, the facility costs would increase by 17% (22% ‘pure’) in the urban district, and by 42% (63% ‘pure’) in the rural district (data not shown). Overall, the planned reforms to the CHF would increase the total cost per member to USD 24 (USD 21 for ‘pure’ costs) in the urban district, and to USD 5 per member (USD 4 for ‘pure’ costs) in the rural district. The cost to revenue ratio would increase to 749% (672%) in the urban district and to 96% (79%) in the rural district with the matching fund.

## Discussion

Obtaining a better understanding of the total administration cost of CBHI and the relative costs of different administrative activities is essential to fully assess the financial sustainability of this approach and its potential contribution to achieving UHC in low-income settings. This is the first study to estimate the economic costs of setting up and administering CBHI and the relative costs of different administration activities.

This article has showed that in Tanzania, the main CHF administrative activities were conducted at facilities and at district headquarters within the government system. The most costly activity at facility level was revenue collection, and more specifically scheme advertising. Advertising in a low-income context where awareness and understanding of the insurance principle is limited and populations may be geographically scattered, requires intensive community engagement and travel—both resource-intensive undertakings (Mathauer and Nicolle 2011). Furthermore, in order to sustain membership levels over time, this activity must be repeated on an ongoing basis. In Tanzania, the community members who are part of the HFGC are supposed to play an important role in advertising the CHF within the community, but were found to undertake relatively limited activities. The study found that in practice health workers are responsible for advertising and registering and enrolling members, consuming up to 30% of a single full-time equivalent staff member per facility. This is somewhat concerning given the limited supply of health workers in rural Tanzania (Kurowski *et al.* 2003; Manzi *et al.* 2012). Furthermore, it means that the success of the CHF depends on the personal commitment and initiative of health workers, as well as their ability to devote time to CHF management. It also restricts the reach of advertising campaigns to patients at the facility, limiting coverage and also increasing the likelihood of adverse selection.

Stewardship and management activities, such as meetings to discuss the CHF and reporting on revenue collected, incurred costs at the facility and district levels. These activities were made more time consuming because of a lack of computerised systems for processing membership information for reporting. At the district level, the CHF co-ordinator is typically fully employed as a health secretary but spends up to 20% of their time on stewardship and management, resulting in a competition between roles (Stoermer *et al.* 2011).

The costs of pooling are currently limited, as funds are simply deposited into a bank account (at facility or district level) and there is no risk equalisation or cross subsidisation. The costs of purchasing were limited to contracting with higher level facilities in one district. However, in more conventional CBHI schemes, the costs of processing claims would need to be included, and these may be substantial in areas with high enrolment (Ranson *et al.* 2006). The CHF, like many other community-based insurance schemes, does not fulfil an active purchasing role (Carrin *et al.* 2005; Bennett *et al.* 1998). While undertaking such a role would increase administrative costs, it would likely reduce the costs of care for beneficiaries, increasing administrative efficiency.

The cost-to-revenue ratio was higher than that reported in most previous studies (Mathauer and Nicolle 2011; Bennett *et al.* 1998; Atim 1998). The cost estimates provided by Mathauer and Nicolle relied on national health accounts data which only consider the financial costs of the scheme, overlooking the economic costs (opportunity costs) to the overall health system (Mathauer and Nicolle, 2011). Other studies relied on published reports from schemes (Bennett *et al.* 1998) or surveys of schemes in specific countries (Atim 1998). In both cases administrative costs were not the main focus of the study, and available data were limited. It has been acknowledged that had the opportunity cost of resources been valued, the administration cost would increase substantially (Bennett *et al.* 1998). This study has shown that opportunity costs of community-based health insurance may be substantial, especially when they rely on government health workers and district managers.

The study suffered from a number of limitations. The study relied on reported estimates of time use by a small sample of health workers and district managers that may not be representative of the rest of the country. Furthermore, we were unable to undertake a time motion study to validate these estimates and ensure that reported time spent reflected actual time spent. The amount of time spent by health workers advertising the scheme is the most questionable, especially in urban areas where coverage was low. However, the reported time spent was relatively consistent across respondents. One may question why health workers would be willing to spend time enrolling members. The CHF premium payments can be used by facilities to address drug stock-outs by purchasing essential drugs and supplies, to undertake minor facility renovations and to pay staff allowances, which may motivate health workers to enrol members. Even if we assume that health workers spent no time at all advertising the CHF (an extreme and unlikely assumption) the cost to revenue still exceeds 30% under most assumptions.

Certain cost items were omitted from the analysis. For example, capital costs were not included, and although the government promotes the distribution of free insurance cards to poor households, these costs were also not included. The costs of administration at higher levels of the system were also not included.

The study offers a number of valuable lessons as to how administrative costs might be contained. It was, for example, shown that facilities with lower case loads were able to achieve a lower cost to revenue ratio than facilities with higher case loads meaning that, as currently structured, the CHF lends itself better (in terms of management cost) to small dispensaries than large health centres. These facilities have lower advertising costs, as they have fewer outpatients, and are more readily able to attain coverage targets among the populations they service. Hence, the scheme CHF could save money by focusing on the lowest-level facilities. Professional management organizations might also be contracted to run management activities more efficiently (Carrin *et al.* 2005).

The costs of the current approach to advertising and marketing are substantial, yet of limited benefit, as qualitative research indicates many community members are unaware of the CHF (Macha *et al.* 2012). Alternative approaches that have been used elsewhere include media campaigns, using community health workers to undertake enrolment, as well as school promotion, for example (Cousineau *et al.* 2011; Meng *et al.* 2011). Such approaches may prove more effective and less resource-intensive compared with undertaking advertising and marketing activities within health facilities. Another way of reducing CHF management costs would be to limit the timing of registration to a specific moment in the year.

This study has shown that the administration of CBHI is not inexpensive, even when the scheme is built into existing government systems. This combined with the limited capacity for revenue collection make it questionable as a financially sustainable and scalable approach to covering the informal sector. Furthermore, studies from settings with different scheme design should be encouraged to deepen our understanding of administrative cost drivers and how best to reduce administrative costs. In parallel, moves to identify innovative and lower cost solutions to provide access to affordable health care for the informal sector should be strongly encouraged.

## Supplementary data

Supplementary data are available at *Health, Policy and Planning* online.

Translated Abstracts

Supplementary Data
