# Supplementary material for: The administrative costs of community-based health insurance: a case study of the community health fund in Tanzania
Source: Health Policy Plan. 2013 Dec 12;30(1):19–27. doi: 10.1093/heapol/czt093 (PMC4287190; doi:10.1093/heapol/czt093)
Supplement: Translated Abstracts [file supp_czt093_czt093_Chinese.pdf]

以社区为基础的医疗保险的管理成本：基于坦桑尼亚一个社区医疗基金的个案研究

以社区为基础的医疗保险的发展被视为一种解决低收入环境中颇具规模的非正规部门的融资手段。然而，较少有数据表明对于这种制度的管理成本。我们检验了经营一个社区医疗基金（CHF）一年的机构成本以及地区级成本，这个社区医疗基金是坦桑尼亚某地区一个志愿性质的医疗保险计划,它为本地区的城乡的非正规部门提供服务。关于资源使用的信息、CHF的成员信息以及税收信息来自于分别地区中两个机构的地区经理和医疗工作者。我们估计了每个 CHF 成员的家庭成本以及成本税收比。在机构层面，征税是成本最高的活动（占全部成本的 78%），其次是管理和经营的成本（占 13%），以及融资的成本（10%）。在区域层面，管理和经营的成本是最主要的活动。每个 CHF 成员家庭的管理成本从每年 3.33 美元到 12.12 美元不等。税收比例成本在 50%到 364%之间变化。从税收结果来看，管理一个 CHF 的成本是相对较高的。在其他收入水平环境，也应该进行类似研究。
