# Supplementary material for: The administrative costs of community-based health insurance: a case study of the community health fund in Tanzania
Source: Health Policy Plan. 2013 Dec 12;30(1):19–27. doi: 10.1093/heapol/czt093 (PMC4287190; doi:10.1093/heapol/czt093)
Supplement: Translated Abstracts [file supp_czt093_czt093_French.pdf]

Les coûts administratifs du régime d'assurance maladie communautaire: étude de cas du fond de santé communautaire en Tanzanie

Accepté le 29 octobre 2013

L'expansion du régime d'assurance maladie communautaire a été proposée comme solution pour financer l'important secteur informel dans des zones à faible revenu. Toutefois, nous n'avons que peu d'éléments sur les coûts administratifs de tels régimes. Nous avons évalué les coûts annuels de fonctionnement du Fond de santé communautaire (FSC) d'un centre au niveau d'un district, qui est une police d'assurance de santé volontaire pour le secteur informel dans une zone rurale et dans une zone urbaine de la même région en Tanzanie. Nous avons obtenu des informations sur les ressources utilisées, sur les adhérents au FSC ainsi que sur les recettes générées grâce à des responsables locaux et au personnel de santé dans deux centres de chacune des zones concernées. Nous avons aussi estimé le coût administratif par ménage adhérent au FSC et le ratio des coûts par rapport aux recettes. Le recouvrement des recettes est l'activité la plus couteuse au niveau d'un centre (78% des coûts totaux), suivi par l'intendance et le management (13%) puis par la répartition des fonds (10%). L'intendance et le management étaient les principales activités au niveau d'un district. Le coût administratif par ménage membre du FSC s'établissait entre USD 3,33 et USD 12,12 par an. Le ratio du coût par rapport aux recettes, lui, variait entre 50% et 364%. Le coût administratif du FSC était relativement élevé par rapport aux recettes générées. Nous devons encourager des études similaires pour des niveaux de revenus différents.

Mots clés : régime d'assurance maladie communautaire, coût administratif, Tanzanie
