# Supplementary material for: The administrative costs of community-based health insurance: a case study of the community health fund in Tanzania
Source: Health Policy Plan. 2013 Dec 12;30(1):19–27. doi: 10.1093/heapol/czt093 (PMC4287190; doi:10.1093/heapol/czt093)
Supplement: Translated Abstracts [file supp_czt093_czt093_Spanish.pdf]

Los costos administrativos del seguro de salud basado en la comunidad: un estudio de caso del fondo de salud comunitario en Tanzania

Josephine Borghi, Suzan Makawia y August Kuwawenaruwa

Aceptado 29 de octubre de 2013

Una expansión del seguro de salud basado en la comunidad ha sido propuesta como una solución de financiamiento para el considerable sector informal en escenarios de bajos recursos. Sin embargo, hay evidencia limitada de los costos administrativos de tales programas. Evaluamos a nivel de instalaciones y de distrito los costos anuales para operar el Fondo de Salud Comunitario (FSC), un esquema voluntario de seguros de salud para el sector informal en un distrito rural y uno urbano en la misma región en Tanzania. Información acerca del uso de recursos, afiliación al FSC e ingresos fue obtenida de los directores de distritos y de los trabajadores de salud de dos instalaciones en cada distrito. El costo administrativo de cada hogar miembro del FSC y la proporción de costo a ingreso fueron estimados. La recolección de ingresos fue la actividad más costosa a nivel de las instalaciones (78% del costo total), seguido por la administración y el manejo (13%) y la acumulación de fondos (10%). La administración y el manejo fueron las actividades principales a nivel de distrito. El costo de administración para cada hogar miembro del FSC osciló entre USD 3.33 y USD 12.12 por año. La proporción de costo a ingreso osciló entre 50% y 364%. El costo de administrar el FSC fue alto relativo a los ingresos generados. Estudios similares en otros escenarios deben ser promovidos.

Palabras Claves
seguro de salud comunitario, costo de administración, Tanzania

MENSAJES CLAVE

- La publicidad y la recolección de ingresos fueron las actividades más intensivas en cuanto a recursos relacionadas con la administración de seguros de salud basados en la comunidad en Tanzania. Estas actividades son llevadas a cabo por los trabajadores de salud de las instalaciones y representan una carga de tiempo sustancial.
- Las actividades de administración y manejo tuvieron el costo más significativo a nivel de distrito. Las actividades representaron una carga de tiempo mayor debido a la falta de sistemas computarizados para el procesamiento de información de afiliación para reportar. Los costos de la acumulación y adquisición fueron mínimos, debido a una falta de ecualización de riesgo o subsidios cruzados y adquisición limitada.
- El costo de administrar el FSC fue alto con relación a los ingresos generados. La proporción de costo a ingresos más que superó la recomendación de 30%.
- Las instalaciones con un menor número de casos lograron obtener una proporción de costo a ingresos menor que las instalaciones con un alto numero de casos lo cual significa que, como está estructurado actualmente, el FSC es más apropiado (en términos de costos de manejo) para los dispensarios pequeños que para los centros de salud grandes.
