# Supplementary material for: The administrative costs of community-based health insurance: a case study of the community health fund in Tanzania
Source: Health Policy Plan. 2013 Dec 12;30(1):19–27. doi: 10.1093/heapol/czt093 (PMC4287190; doi:10.1093/heapol/czt093)
Supplement: Supplementary Data [file supp_30_1_19_v2_index.html]

The administrative costs of community-based health insurance: a case study of the community health fund in Tanzania — The administrative costs of community-based health insurance: a case study of the community health fund in Tanzania — Supplementary Data 

# The administrative costs of community-based health insurance: a case study of the community health fund in Tanzania

## Supplementary Data

files

**Files in this Data Supplement:**

- Supplementary Data - docx file
