# Supplementary material for: The administrative costs of community-based health insurance: a case study of the community health fund in Tanzania
Source: Health Policy Plan. 2013 Dec 12;30(1):19–27. doi: 10.1093/heapol/czt093 (PMC4287190; doi:10.1093/heapol/czt093)
Supplement: Supplementary Data [file supp_czt093_Annexes_edit.docx]

**Annexes**

*Annex 1: Assumptions*

Costs spanning multiple years such as the CHF card which can be used for up to 5 years, were annualised over the relevant time period. Start up costs were also annualised: the costs of training health facility governing committee members were annualised over a three year period, as these committees are elected to run for three years. The costs of setting up the service agreement with referral facilities were also annualised over a three year period, on the assumption that such contracts would need to be revisited every three years.

When estimating the time spent by HFGC and CHSB members in meetings we only included the time costs of those meetings where the CHF was discussed. As these meetings typically cover a range of issues, the proportion of time related to CHF was estimated by considering the total number of agenda items (i.e. if there were six agenda items including a discussion of the CHF, then the time allocated to the CHF would be 1/6^th^ of the total meeting duration).

In the case of activities related to CHF membership (i.e. enrolling members), or to the number of outpatient visits (publicising the CHF), the average time spent was multiplied by the number of CHF members or outpatient visits in the facility.

Annex 2

Table 1A: Unit Costs of Key Resource Items in USD

| **Resource item** | **Unit cost (annualised cost)** | **Assumption** |
| --- | --- | --- |
| **Staff salary per hr** |  |  |
| CHF coordinator | 3.44 |  |
| Doctor | 2.38 |  |
| Nurse | 1.76 |  |
| HFGC community member | 0.23 |  |
| CHSB member | 3.44 |  |
| **Allowances** |  |  |
| HFGC dispensary meetings | 3.56 | 3.16 allowances per person plus 3.16 for group refreshments |
| HFGC health centre meetings | 9.89 | 9.49 allowances per person plus 3.16 for group refreshments |
| CHSB meetings | 62.08 | Per person. |
| Day out allowance for facility supervision | 11.08 |  |
| **Transport to deposit funds** | 2.22 | Range from 1.27 to 3.16 |
| **Supplies** |  |  |
| CHF card | 0.63 (0.13) |  |
| Plastic case for CHF card | 0.95 (0.19) |  |
| Counter book | 1.58 (0.53) |  |
| Receipt | 0.02 | 1.58 per book, 100 receipts per book |
| Photocopy | 0.06 |  |
| Paper | 0.03 |  |
| Pen | 0.13 |  |

Table 2A: Quantities of Resource Inputs by Activity and Nature of Cost (Fixed – Variable) – Average for All Facilities Across Both Districts

|  | **Resource intensity per activity: Time** | **Annual time spent in hrs** | **Other resources** | **Nature of cost** |
| --- | --- | --- | --- | --- |
| Advertising, marketing:  Individual | 2.5 minutes spent with each uninsured outpatient for all facilities | 472 (101-1079) |  | Variable |
| Advertising, marketing: Group | 10 minutes spent with varying frequency (minimum never, maximum once per week) | 4.5 (0-9 hrs) |  | Fixed |
| Advertising, marketing: Community | **Ad-hoc meetings:**  Average 2 people (1-3), for 38 mins (30-45) 7 times per year (2-12).  **Ward meetings:**  Village meetings: once per year 17.5 minutes (min 15, max 20)  Once per quarter 1 person for 15 minutes. | 9 (2-27) |  | Fixed |
| **Total Advertising, marketing** |  | **485.5**  **(103-1115)** |  |  |
| Registering and enrolling members | 5 minutes per CHF member | 19 (9-33) | 1 counter book for 3 years (max: 1 book per year)  1 receipt per member  1 card per member for 5 years  1 plastic case per member for 5 years  1 pen per year | Variable |
| Fund management | 10 times a year (min 4 times, max 12).  4.4 hrs (min 2 hrs max 8hrs). | 44 (8-96) | 3300 TZS (1950 – 4650 TZS) in transport costs  1 photocopy  1 receipt that funds were received | Fixed |
| Meetings: Facility level | **HFGC:** 1.75 times per year (min 1 – max 2) 1.67 hrs (min 30 mins – max 3 hrs). Attended by 8 people  **WDC:** 4 times per year; 22 mins (min 10 mins, max 45 mins). Attended by 7 people.  **Village: meetings:** 4 times per year for 10 mins. Attended by 7 people. | 23 (4-48)  5(10-21)  5 | Allowances for HFGC members. Allowance apportioned based on % of meeting dedicated to CHF.  63% (25%-100%) | Fixed |
| Meetings: District level | 68 minutes (min 45 mins, max 1.5 hrs). Attended by 8 people. Once per year. | 9 (6-12) | Allowances for CHSB members. Allowance apportioned based on % of meeting dedicated to CHF. 40% (30%-50%) | Fixed |
| **Total Meetings** |  | **42 (25-86)** |  |  |
| Reporting: Facility level | Once per month, 2.5 hours (min 1 hr - max 3.5 hrs)  Once per quarter , 2.25 hrs (min 1.5 hrs, max 3 hrs) | 30 (12-42)  9 (6-12) |  | Fixed |
| Reporting: District level | 4 times per year; 9 hours (min 8, max 10 hrs) | 36 (32-40) |  | Fixed |
| **Total reporting** |  | **75 (50-94)** |  |  |
| Supervision | **CHMT:** 4 times, 1.2 hrs (min 20 mins, max 2 hrs)  **Independent:** 1 time per month, 8 hrs | 4.8 (1.3-8)  96 |  | Fixed |
| Training | 4 people for 3 days entire district – urban  4 people for 0.5 day per ward (rural) |  | Not specified. | Fixed |
| Service agreement | 1 person for 5 full days | 40 |  | Fixed |
